# Characteristic differences between the promoters of intron-containing and intronless ribosomal protein genes in yeast

**DOI:** 10.1186/1756-0500-1-109

**Published:** 2008-10-29

**Authors:** Jing Zhang, Martin Vingron, Stefan Roepcke

**Affiliations:** 1 Max-Planck Institute for Molecular Genetics, Berlin, Germany; 2 Department of Statistics, Yunnan University, Kunming, PR China; 3 Nycomed GmbH, Konstanz, Germany

## Abstract

**Background:**

More than two thirds of the highly expressed ribosomal protein (RP) genes in *Saccharomyces cerevisiae *contain introns, which is in sharp contrast to the genome-wide five percent intron-containing genes. It is well established that introns carry regulatory sequences and that the transcription of RP genes is extensively and coordinately regulated. Here we test the hypotheses that introns are innately associated with heavily transcribed genes and that introns of RP genes contribute regulatory TF binding sequences. Moreover, we investigate whether promoter features are significantly different between intron-containing and intronless RP genes.

**Results:**

We find that directly measured transcription rates tend to be lower for intron-containing compared to intronless RP genes. We do not observe any specifically enriched sequence motifs in the introns of RP genes other than those of the branch point and the two splice sites. Comparing the promoters of intron-containing and intronless RP genes, we detect differences in number and position of Rap1-binding and IFHL motifs. Moreover, the analysis of the length distribution and the folding free energies suggest that, at least in a sub-population of RP genes, the 5' untranslated sequences are optimized for regulatory function.

**Conclusion:**

Our results argue against the direct involvement of introns in the regulation of transcription of highly expressed genes. Moreover, systematic differences in motif distributions suggest that RP transcription factors may act differently on intron-containing and intronless gene promoters. Thus, our findings contribute to the decoding of the RP promoter architecture and may fuel the discussion on the evolution of introns.

## Findings

### Background

#### Hypothesis and Work Plan

In this study, we investigate three hypotheses. First, introns are innately associated with heavily transcribed genes [1]. Second, introns of RP genes carry regulatory TF binding motifs [2-4]. And third, promoter features like Rap1 binding sites or the GC base profile are significantly different between intron-containing and intronless RP genes [5]. To this end, we construct three promoter sets of intron-containing and intronless RP genes, and of non-RP lowly expressed intron-containing genes [6-10]. We compare mRNA expression levels, transcription rates, 5'UTRs, and base compositions around the TSS. Additionally, we scan the promoter sequences for potential binding sites of several transcription factors and investigate their frequencies and localizations relative to the TSS. Finally, we test effects of identified promoter features on RP gene expression by linear regression analysis. For a further background we refer to Additional file 1.

#### Gene Structure, Expression and Transcription Rate

**Figure 1:**
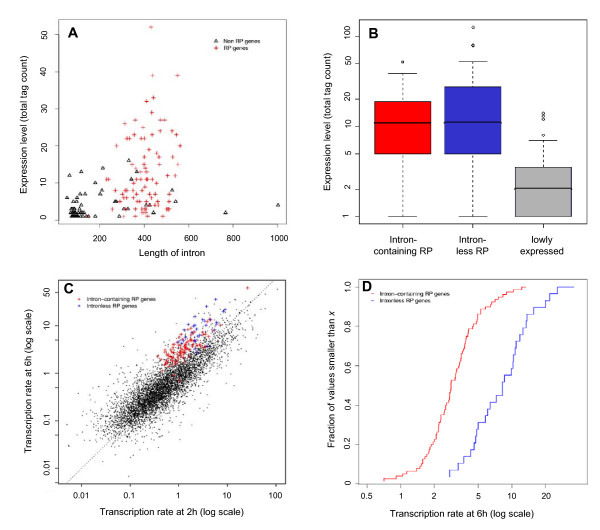
**Gene expression and intron lengths; transcription rates**. The upper panels A and B show expression levels measured by 5'SAGE [12] and the lower panels C and D show transcription rates measured by a transcription run-on assay [11]. (A) The expression level (5'-SAGE tag count) is depicted versus the intron length. (B) The distributions of the expression levels (5'-SAGE tag counts) are depicted in boxplots for each of the gene sets. (C) The transcription rates (TR) measured two and six hours after glucose-galactose shift are plotted. Measurements of all non-RP genes are depicted as black points. The diagonal line marks the identity. (D) The empirical cumulative distribution functions of TR at six hours of the intron-containing (red) and intronless (blue) RP genes are depicted. For example, a blue point (*x*,*y*) represents the fraction *y *of the intronless RP genes that have smaller TR than *x*.

The average mRNA abundance of intron-containing and intronless RP genes is not significantly different when measured with SAGE (Fig. 1B, F-test *p*-value: 0.166) [see Materials and Methods in Additional file 1]. We only present the results for the SAGE data set but the analysis of microarray data sets leads to qualitatively similar results. Note that we use the terms mRNA abundance and expression level synonymously to designate the mRNA level averaged over large numbers of cells in different experimental conditions. In contrast, the transcription rate, which was measured directly by a genome-wide transcription run-on assay [11], is systematically higher in intronless RP genes in yeast cells recovering from a glucose-galactose shift (F-test *p*-value: 1.479e-09; Fig. 1C, D) [see Methods in Additional file 1]. This may reflect the additional costs for transcribing the intronic sequence and splicing of the pre-mRNA. And one could speculate that mRNA stability counter balances the faster mRNA production of intronless RP genes. Note that the average mRNA abundance is a summary value of yeast cells in different states, which are not compatible with the conditions of the transcription run-on assay. We conclude that introns are not necessary for RP genes, and hence for yeast genes in general, to be highly expressed. Note that these data also show very nicely the concerted induction of transcription of virtually all RP genes compared to most other genes at six hours after the glucose to galactose shifting (Fig. 1C).

In order to obtain more accurate information about the positioning of potential regulatory motifs, we incorporate transcription start site (TSS) predictions derived from 5'SAGE experiments [12]. In a recent study, TSSs were determined for the majority of yeast genes and there is good concordance between the results of the two studies [[9,12], see Additional file 1]. We use the predictions of the 5'SAGE study throughout this work. For 90 of the 100 intron-containing and 33 of the 37 intronless RP genes, we find TSS predictions in this data set. We restrict further analyses to this subset of 123 genes. Traditionally, for the study of the relative localization of transcription factor binding sites (TFBS), the translation start codon ATG is taken as a surrogate for the TSS, which can be rather inaccurate especially for genes that contain an intron in their 5'UTR (leader intron). We select an additional set of 35 lowly expressed intron-containing genes that are also present in the 5'SAGE data set in order to contrast our results for the RP genes [[13], Fig. 1B, see Additional file 2].

**Figure 2:**
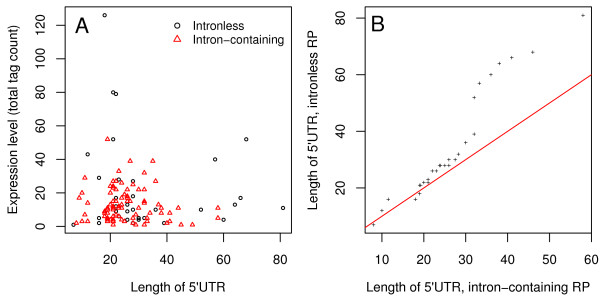
**Dependence of expression level and 5'UTR length**. (A) Expression levels (5'-SAGE tag counts) are depicted in relation to the lengths of the 5'UTRs using different symbols for the two RP gene sets. (B) The length distributions of the 5'UTRs of intron-containing (*x*) and intronless (*y*) RP genes are compared in a Quantile-Quantile plot. For example, the 0.9-quantile of *x *is the value (5'UTR length) *k *such that 90% of all *x*-values are smaller than *k*. The quantiles of the two distributions are plotted against each other to identify systematic deviations. The diagonal line marks equality.

We estimate the lengths of the 5'UTRs as distance between the translation start codon (ATG) and the predicted TSS from the 5'SAGE experiments, excluding introns. There is no strong dependence of the UTR length on the mRNA expression level, although the three most highly expressed genes, RPL38, RPL41A and RPL41B have short UTRs (Fig. 2). Although the 5'UTRs of intronless RP genes are significantly longer, the distributions are not separated or, in other words, some intron-containing genes also have relatively long 5'UTRs (F-test *p*-value: 0.00761). The most pronounced difference is observed for the longest 5'UTRs, which may form a special group. To investigate this further, we used the Vienna package to compute the folding free energy (Δ*G*) of the first 50 bases of each RP mRNA including the 5'UTR [14]. Among the seven intronless genes with longest 5'UTR sequences, we find the five most stable secondary structure elements, which suggests a role in the regulation of translation [15]. Moreover, we find a significant negative correlation between the folding free energy of the 5'-UTR and the mRNA abundance (correlation coefficient: -0.3, *p*-value: 0.00205) but not the transcription rate (correlation coefficient: 0.06, *p*-value: 0.5605).

#### Distribution of Rap1 Binding Motifs

For factors that are known to regulate RP gene transcription and those that have been predicted by genome-scale experiments, we select position weight matrices (PWM) to represent the binding specificity and scan the region from 600 bp upstream of the TSS to 600 bp downstream of all the genes of our three sets for potential binding sites using T-Reg [see Additional files 1, 3, 4, 5, 6, 7, 8, 9 for methods and for more findings].

**Figure 3:**
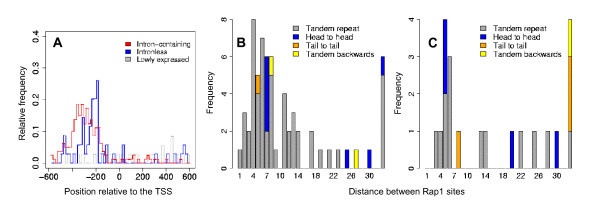
**Rap1 binding sites – location and duplicate frequencies**. Chart (A) depicts the distribution of the occurrence of the Rap1 motif in the three different promoter sets. (B) and (C) depict the relative position or distance and the orientation of the duplicate Rap1 sites (B) for intron-containing RP genes, and (C) for intronless RP genes. In the last bin denoted by "<100" all duplicated Rap1 sites of distances greater than 30 and smaller than 100 are subsumed.

We scan our promoter sets for Rap1 binding motifs similar to Lascaris and colleagues using the PWM MR2 with consensus string WACAYCCRTAACATY [16]. As the general findings regarding position and orientation of Rap1 binding motifs in RP promoters are confirmed by our analysis, we focus on differences between intron-containing and intronless genes. We predict potential binding sites in all intron-containing genes except *RPS22B*. In contrast, we did not detect Rap1 sites in six of the 33 intronless genes (see tables in Additional file 1). Moreover, although in both sets of genes the binding sites are located mainly in the expected region between positions -500 and -160, there are characteristic differences. In intronless RP genes, the Rap1 sites occur narrowly distributed around position -220 (Fig. 3a). In intron-containing genes, the sites are distributed over a broader range, mainly between positions -380 and -300, which is further upstream of the TSS (Fig. 3a). Former studies have demonstrated that Rapl binding sites mostly occur in pairs and in a preferred orientation [16,17]. According to our analysis, 74 of 90 intron-containing genes with predicted TSS have pairs of Rap1 sites, of which 64 are spaced less than 30 bp (Tab. 1 in Additional file 1, Fig. 3b). By spacing, we mean the number of bases in between the two sites. In one gene, the sites are 88 bp apart and in ten genes, more than 100 bp, which we consider as abnormal pairs. The preferential spacing is two to six bp. Most commonly, the two Rap1 sites occur in tandem according to the consensus given above and second most in head-to-head orientation, which together account for 95% of the cases (Fig. 3b). In this aspect, there are no big differences between intron-containing and intronless RP genes (Fig. 3c). In the whole RP promoter set, we identify 13 duplicate Rap1 sites with short spacing (<30 bp) and single Rap1 sites in three genes, in addition to the findings of Lascaris and colleagues [16]. Because the newly identified Rap1 sites occur in the proper location and orientation, we are confident that our T-Reg method produces specific but sensitive predictions.

#### Distribution of IFHL

In contrast to Fhl1 and Sfp1 motifs (see Additional file 1 for details), the IFHL motif occurs quite differently in the two RP promoter sets and is barely present in promoters of lowly transcribed genes (Additional file 9). We identify 69 instances in 40 intron-containing genes between positions -400 and -150 (Additional file 1, 9). Nine intronless genes contain IFHL motifs, five of which are in upstream promoter regions comparable to intron-containing genes (position -400 to -150). The IFHL motif is preferentially located downstream of the Rap1 sites within a distance of 50 bp. Sometimes the two motifs overlap. This is in accordance with previous results [18]. Furthermore, the IFHL motifs in the upstream region of 24 intron-containing genes occur in duplicate within a distance of less than 100 bp (Tab. 1 in Additional file 1). Other than the mentioned differences in the positioning of the Rap1 motifs, the distribution of IFHL displays the most pronounced differences between intron-containing and intronless RP genes (Chi-squared test p-value: 0.04124).

## Conclusion

Two findings of our analysis argue against the direct involvement of introns in the regulation of transcription of the highly expressed group of ribosomal protein genes. First, we show that introns are not necessary for RP genes in yeast to be heavily transcribed. Second, introns of RP genes are not enriched in binding motifs of known or putative RP transcription factors. Furthermore, we test the effect of promoter features on expression level and transcription rate by linear regression analysis. This is important because at present we cannot explain the large variety of transcription rates and of expression levels of the highly and coordinately expressed RP genes. We find that the most significant features are, for the transcription rate, the presence of introns and for the expression level, the folding free energy of the 5'-terminal sequence. Our results help to decipher the RP promoter architecture towards a prediction of transcription rates based on the presence and strength of sequence features.

## Competing interests

The authors declare that they have no competing interests.

## Authors' contributions

JZ carried out many of the analyses and prepared data for publication. MV provided initial ideas and concepts and helped writing the manuscript. SR carried out analyses and wrote the manuscript.

## Supplementary Material

Additional File 1 Additional background, findings, materials and methods. Additional background, findings, methods and the relevant references are presented. Click here for file

Additional File 2 TSS positions. The TSS positions of the sample genes (intron-containing, intronless RP and lowly expressed intron-containing) referring to Zhang et al (2005) are listed. Click here for file

Additional File 3 RP gene transcription factors. Transcription factors that are enriched on RP gene promoters in ChIP-Chip experiments. Click here for file

Additional File 4 Weight matrices for TFBS search. This file contains the weight matrices for the transcription factors that have been used for promoter scans for potential binding sites. Click here for file

Additional File 5 Promoter regions of RPS29A and B. A snapshot from the Genome Browser illustrates a typical the structure of RP gene promoters. Click here for file

Additional File 6 Regression table. This file contains the data table that was used for linear regression analysis. Click here for file

Additional File 7 Genome wide Arr1 motif scan. The yeast ORF's (SGD) including 1000 bases upstream of the ATG are scanned for Arr1 binding motifs. Click here for file

Additional File 8 Profile of GC base content. The profile of GC base content is specifically optimized in the highly expressed RP genes. Click here for file

Additional File 9 Motif hit distributions. Distributions of binding site motifs for several transcription factors. Click here for file
