# Supplementary material for: Characteristic differences between the promoters of intron-containing and intronless ribosomal protein genes in yeast
Source: BMC Res Notes. 2008 Oct 29;1:109. doi: 10.1186/1756-0500-1-109 (PMC2585575; doi:10.1186/1756-0500-1-109)
Supplement: Additional File 1 — Additional background, findings, materials and methods. Additional background, findings, methods and the relevant references are presented. [file 1756-0500-1-109-S1.doc]

Additional file 1

Characteristic Differences Between the Promoters of Intron-Containing and Intronless Ribosomal Protein Genes in Yeast

**Authors:** Jing Zhang, Martin Vingron, Stefan Roepcke

[Background 2](#__RefHeading___Toc212369926)

[Additional Findings 4](#__RefHeading___Toc212369927)

[Materials and Methods 9](#__RefHeading___Toc212369928)

[Extended Bibliography 12](#__RefHeading___Toc212369929)

[Tables 16](#__RefHeading___Toc212369930)

[Figure Legends 21](#__RefHeading___Toc212369931)

## Background

The transcriptional regulation of ribosomal protein (RP) genes is being studied extensively in baker’s yeast *Saccharomyces cerevisiae* [1-4]. RP genes are attractive for the investigation of fundamental gene regulatory mechanisms for several reasons [5]. First, they are part of an essential cellular machinery present in each living organism. Second, the genes are highly and coordinately expressed because their products are needed in large and equimolar amounts in proliferating yeast cells. Third, under nutritional shortage or other stress conditions, RP gene transcription is abruptly shut down and quickly up-regulated if optimal growth conditions are restored. Fourth, more than half of the RP genes contain introns, which is contrary to the majority of yeast genes.

The genome of *Saccharomyces cerevisiae* contains at least 137 ribosomal protein genes [6]. In accordance with the fact that these genes are coordinately expressed, the general regulatory factors Rap1 and Abf1 have been identified to be involved in their regulation [2]. In particular, Rap1 binding sites are found in virtually all RP promoters, mostly in pairs and at a preferred position relative to the transcription start site (TSS). Moreover, Rap1, Reb1, and Abf1 can function synergistically [7-9] and for some regulated genes it has been shown experimentally that they can functionally replace each other [10, 11]. Furthermore, T-rich regions proximal to Rap1 sites have been shown to be required for proper regulation of RP gene transcription [1].

Recent genome-wide chromatin immuno-precipitation experiments (ChIP-Chip) have identified Ifh1, Fhl1 and Sfp1 as important additional regulators of RP gene transcription [4]. The transcription factor Ifh1 plays a central role in the gene regulation of RP genes via its interaction with the forkhead-associated domain of Fhl1 [4, 12-15]. Fhl1, in turn, is believed to bind the promoter sequence directly. Based on ChIP-Chip experiments, the novel IFHL motif has been derived from RP promoter sequences that are specifically bound by Ifh1 and Fhl1 in addition to Rap1 [4]. However, it is not known whether this motif actually characterizes the binding specificity of the Ifh1/Fhl1 complex. Another critical factor for the transcription of yeast RP genes is Sfp1, for which it is not clear whether it binds the DNA directly. Sfp1 strongly influences cell size and might influence the transcription of RP genes via Fhl1 and Ifh1 [16].

In addition to these factors, genome-wide studies of co-regulated genes using microarray experiments and of promoter binding of transcription factors using ChIP-Chip coupled with motif discovery have resulted in a number of new potential RP gene regulators and over-represented regulatory motifs [17-21].

Recent studies of human RP promoters by other groups and by us have shown that accurate knowledge about the start site of transcription may foster the discovery of novel regulatory motifs and of characteristics of the base composition [22-24]. For yeast, Zhang and Dietrich used a SAGE based technology to obtain thousands of 15-17 bp long snippets from the 5’-end of capped mRNA (5’SAGE), which resulted in estimates of the transcription start sites of more than 2000 yeast genes [25]. In a complementary study, Miura and colleagues determined large-scale full-length cDNA by applying a vector capping method and mapped start sites for 3599 ORFs [26].

Although in yeast, introns are found in less than 5% of the approximately 6000 protein-coding genes, 100 of the 137 RP genes contain introns [6, 26-29]. The extra costs that come with keeping introns in the highly expressed RP genes are likely to be balanced with selective advantages. One possible mechanism comes from the observation that some ribosomal proteins can bind their own pre-mRNA and thereby influence its own expression level post-transcriptionally [5]. A second mechanism can be derived from the fact that downstream regions like introns and even coding sequence have been shown to contain regulatory sequences [30-32]. In a previous bioinformatic analysis we demonstrated that introns of highly expressed genes harbor over-represented sequence patterns that could potentially be involved in transcriptional regulation in yeast [33, 34].

## Additional Findings

### Base Composition Profile

The base composition of DNA sequences around the TSS displays a characteristic pattern for the highly expressed RP genes [Additional file 8a,b]. The average GC content upstream of position –300 and downstream of +30 is about 35-38%. It is increased between –300 and –160 and drops quickly around –80. Shortly before the TSS it is minimal and increases to the background level until the approximate position +30. This is consistent with previous calculations and agrees with the well-documented observation that transcription often starts in regions of lower levels of hydrogen bonding flanked by regions of higher levels [25, 35, 36]. In contrast, lowly transcribed intron-containing genes do not show this pattern [Additional file 8c]; the GC content is slightly lower from immediately upstream of the TSS until about 100bp downstream of the TSS. Although the GC profiles of the intron-containing and of the intronless RP genes are overall similar, there are slight differences between the two sets. First, the GC content of introns is lower than that of the reading frames, about 35% versus 40% on average. The usually short and mostly non-coding first exons do not influence the GC content considerably [37, Additional file 8]. Second, the increase of the GC content in the –300 to –160 bp region appears to be stronger in intron-containing genes. Whether the pronounced base profile mainly helps the machinery to recognize the promoter or facilitates the DNA duplex melting at the transcription initiation site is not clear to our knowledge. It is even conceivable that the fine-tuning of transcription rates is accomplished via the adaptation of the energy needed for promoter melting and not by the abundance or binding characteristics of transcription factors. This could be tested experimentally by interchanging promoter sequences of differentially expressed RP genes.

### Distribution of Rap1 Binding Motifs

There are single Rap1 site predictions downstream of the TSS, which, according to Lieb and colleagues, may not be bound by Rap1 [38]. Since Rap1 sites are necessary for the high expression of RP genes, we tried to correlate the Rap1 site occurrences and the mRNA expression level. We do not find any significant relationships. In particular, several genes with only one Rap1 site are nonetheless highly expressed. The only possible correlation is that the occurrences of the Rap1 sites for the three intron-containing genes *RPS22B*, *RPS14B* and *RPS9A* with lowest expression levels show abnormal distributions. *RPS22B* has no Rap1 sites, and *RPS14B* and *RPS9A* have duplicate Rap1 sites but the spacing is more than 200 bp.

### Distribution of Fhl1, Sfp1 Binding Motifs

The binding of the transcription factors Fhl1 and Sfp1 to promoter DNA has not been characterized in detail. Therefore, we use the PWMs that where generated by pattern finding from genome-wide ChIP-Chip experiments to predict binding sites in our promoter set [19]. These PWMs resemble each other and, to a lesser extent, the one of Rap1. The weight matrices for Fhl1 and Sfp1 are not very specific; consequently, sites are predicted in almost all RP genes as well as in lowly-transcribed genes [Additional file 9]. The distributions of the predicted binding sites of Sfp1 and Fhl1 show some enrichment between positions –500 and –160, which is the region of the Rap1 binding sites. Furthermore, Fhl1 binding sites are enriched around the TSS in both RP gene sets and even in lowly transcribed genes, and around position +450, which resides mainly in coding sequences of intronless genes. A caveat with automatic motif discovery in sequence sets that are enriched in RP promoters is that most of them have duplicate Rap1 sites. As a result, in most cases, its motif is discovered first. Therefore, based on our findings, we believe that, at best, only a fraction of the predicted sites are actually Fhl1 or Sfp1 binding sites.

### Distribution of Abf1 and Reb1 Binding Motifs

The annotation of the promoter regions for potential binding sites of the global regulatory proteins Abf1 and Reb1 using the PWMs from Harbison and colleagues reveals that there is not such a strong preferential localization as per the Rap1 motif [Additional file 9]. We find more predicted Abf1 binding sites in the upstream region of intronless genes compared to the intron-containing ones, with two stronger peaks at approximate positions –160 and –380. The strongest signal of the predicted Reb1 sites is found in the promoters of intronless genes also at the approximate position –160. In intron-containing genes, however, more predicted binding sites for both Abf1 and Reb1 occur downstream of the TSS compared to upstream regions (Tab. 1). The majority of downstream Abf1 sites is situated in introns and some are found in the coding sequences (CDS). For Reb1, however, the majority of predicted downstream sites is situated in the coding sequences and not in introns, which could support another well-documented function of Reb1, the termination of transcription [39, 40]. In fact, we detected Reb1 motifs located in 3’UTRs or coding sequences in almost all of the genes, both intron-containing and intronless.

Our next question is whether Abf1, Reb1 or IFHL binding proteins are likely to functionally compensate for Rap1 binding sites. We consider Abf1 and Reb1 sites, IFHL occurrences that are located between positions –520 and –140, as potentially compensatory and find that the great majority of the RP promoters with none or only a single Rap1 site have a potential binding site for Abf1. Five genes without Abf1 motifs have Reb1 or IFHL motifs in addition to one Rap1 site (Tab. 1,2). In six RP genes we only find one potential binding site in the –520/–140 region and in the promoter of *RPP2B* we could not find a single motif. Although we don’t have statistical proof, our findings support the view that the highly efficient RP promoters of yeast contain a pair of binding sites for general regulatory factors, mainly Rap1, in a preferred region more than 140 bp upstream of the TSS, and that Abf1, Reb1 and IFHL binding proteins could functionally compensate for missing Rap1 binding sites.

### Distribution of Arr1, Yap1 and Rpn4 Binding Motifs

In addition, we examined the distributions of potential binding sites for the transcription factors Arr1, Yap1 and Rpn4 because, according to the YEASTRACT database, they are associated with the highest number of RP genes [41]. Arr1 and Yap1 belong to the same family of bZIP transcription factors and are known to be involved in the process of arsenite detoxification [20, 42]. In contrast to the other factors, for Arr1 there is neither a weight matrix in TRANSFAC nor in the Harbison set. We therefore used the two sequences TTAATAA and TTACTAA to search for potential binding sites in our promoter sets (Wysocki, et al., 2004 and references therein). The sites predominantly occur at the approximate position +400 in intron-containing and in intronless genes [Additional file 9]. In the intron-containing genes, the motif is found to co-locate with the intron branch points. Since the core consensus TACTAAC of the branch point is very similar to the Arr1 binding preference, the motif hits around position +400 could be false positive predictions. Astonishingly, there are Arr1 motifs at about the same location in intronless genes. We checked these five genes in the data of Kellis and colleagues where the authors identified a number of previously unknown introns [43]. However, there is no evidence that they contain missed introns. Four of the sites with the pattern TTACTAA are situated in the coding sequences and one is in the 3’UTR. Our data suggests that the Arr1 motif downstream of the TSS is of functional importance for the transcriptional regulation of RP genes. Extending this analysis to the whole genome, we scanned all ORF’s from SGD, including 1000 bp upstream of the ATG start codon, for Arr1 motifs [44]. As expected from the dominating role of RP genes, there is enrichment of Arr1 motifs between position +300 and +500 among the intron containing genes (71 hits) [Additional file 7]. In contrast, although we find 323 Arr1 motifs between positions +300 and +500 in intronless genes, there is no specific enrichment as in the RP genes. The PWM of Yap1 is relatively unspecific, and there are consequently many motif hits in the promoter sets. The strongest signal is found around the TSS in both the intron-containing and the intronless genes [Additional file 9]. Downstream of the TSS, there are many more Yap1 sites predicted in intron-containing RP genes compared to intronless RP genes and intron-containing but lowly expressed genes. However, this might be an artifact of the low specificity of the Yap1 weight matrix and the higher GC-content in coding regions compared to intronic sequence. Rpn4 is well known as a key transcriptional regulator of genes that code for proteasomal subunits. There is no preferred localization of the Rpn4 motif and the general occurrence is quite similar in the two RP promoter sets and in our set of lowly expressed genes [Additional file 9].

## Materials and Methods

The DNA sequences of the RP genes, including 600 bp upstream of the TSS, were obtained from the Ribosomal Protein Gene Database [6], [http://ribosome.miyazaki-med.ac.jp](http://ribosome.miyazaki-med.ac.jp/). Throughout the paper, we consider the region that spans 600 bp upstream to 600 bp downstream of the TSS. Information about the introns, including their length and the accurate positions of their splice sites were obtained from YIDB and a recent publication of Miura and colleagues [26, 45]. To investigate the structure of single genes with respect to its evolutionary conservation, we use the UCSC Genome Browser [46].

Zhang and Dietrich determined sequence tags from the 5’-end of transcripts for over 2000 yeast genes by the recently developed technique 5’SAGE, including 123 of the 137 RP genes [25]. From this data set we assess the TSS of each gene, which is the position of the highest tag count. For genes with two or more positions with equal tag counts, we choose the one that is located closest to the typical distance from the start codon ATG [Additional file 2]. This yields TSS predictions for 90 intron-containing and 33 intronless RP genes. A recent large-scale study of full-length cDNA’s provides a complementary set of TSS estimates. Because for most genes more than one TSS is observed, the authors provide the most proximal and the most distal upstream start site, which defines the start site region. For all but 12 genes, the TSS predicted from the 5’SAGE study (STSS) is located in the start site region of Miura and colleagues. Among those are four genes for which the STSS starts one base pair further downstream from the region and three genes for which it starts two or three bp downstream. RPL20A has been newly identified as intron-containing and has been updated in our original data set. This leaves the four genes RPS4A, RPL9B, RPL39B and RPL12B with larger discrepancies in the TSS prediction. As estimates for the average gene expression levels, we adopt the data of two microarray studies and the total 5’SAGE tag counts [18, 25], <http://web.wi.mit.edu/young/expression>. We use the data of Garcia-Martinez and colleagues at the time points *t2* and *t3* (2h and 6h after glucose-galactose shift) as estimates for transcription rates in recovering yeast cells after a global gene expression pause [47].

Transcription factors that are reported to regulate RP genes are collected from the database YEASTRACT, which is accessible at [http://www.yeastract.com](http://www.yeastract.com/) [41, Additional file 3]. The binding specificity of a transcription factor is represented by position weight matrices (PWM) and for scanning the RP promoter regions for potential binding sites we use T-Reg Comparator with a cutoff of *p* < 0.001 [48, 49]. For Rap1 we use the less stringent but more sensitive PWM MR2, which was proposed by Lascaris and colleagues [50]. For the other factors, we adopt the PWMs constructed by Harbison and colleagues [19], which can be obtained at (<http://fraenkel.mit.edu/Harbison/release_v24/final_set/Final_Motifs/>). This website provides PWMs generated by six motif discovery methods under several conditions. When available, we use AlignACE PWMs under the rich medium condition YPD. For Sfp1 we adopt the MEME-c matrix under the condition SM [Additional file 4].

For statistical analyses and the generation of plots we use the software package R ([http://www.r-project.org](http://www.r-project.org/)). For regression and correlation analysis we compile a data table containing expression levels, transcription rates and all the promoter features and apply the R-function *lm* [see Additional file 6]. If not stated otherwise we use the summary method for the R-function lm to apply an F-test and derive p-values. The values of the following parameters are log-transformed for each analysis: total 5’SAGE tag count, 5’UTR length, average expression level, transcription rate.

## Extended Bibliography

1. Goncalves PM, Griffioen G, Minnee R, Bosma M, Kraakman LS, Mager WH, Planta RJ: **Transcription activation of yeast ribosomal protein genes requires additional elements apart from binding sites for Abf1p or Rap1p**. *Nucleic Acids Res* 1995, **23**(9):1475-1480.

2. Lascaris RF, Groot E, Hoen PB, Mager WH, Planta RJ: **Different roles for abf1p and a T-rich promoter element in nucleosome organization of the yeast RPS28A gene**. *Nucleic Acids Res* 2000, **28**(6):1390-1396.

3. Planta RJ, Goncalves PM, Mager WH: **Global regulators of ribosome biosynthesis in yeast**. *Biochem Cell Biol* 1995, **73**(11-12):825-834.

4. Wade JT, Hall DB, Struhl K: **The transcription factor Ifh1 is a key regulator of yeast ribosomal protein genes**. *Nature* 2004, **432**(7020):1054-1058.

5. Warner JR, Vilardell J, Sohn JH: **Economics of ribosome biosynthesis**. *Cold Spring Harb Symp Quant Biol* 2001, **66**:567-574.

6. Nakao A, Yoshihama M, Kenmochi N: **RPG: the Ribosomal Protein Gene database**. *Nucleic Acids Res* 2004, **32**(Database issue):D168-170.

7. Packham EA, Graham IR, Chambers A: **The multifunctional transcription factors Abf1p, Rap1p and Reb1p are required for full transcriptional activation of the chromosomal PGK gene in Saccharomyces cerevisiae**. *Mol Gen Genet* 1996, **250**(3):348-356.

8. Yarragudi A, Miyake T, Li R, Morse RH: **Comparison of ABF1 and RAP1 in chromatin opening and transactivator potentiation in the budding yeast Saccharomyces cerevisiae**. *Mol Cell Biol* 2004, **24**(20):9152-9164.

9. Scott EW, Baker HV: **Concerted action of the transcriptional activators REB1, RAP1, and GCR1 in the high-level expression of the glycolytic gene TPI**. *Mol Cell Biol* 1993, **13**(1):543-550.

10. Goncalves PM, Maurer K, van Nieuw Amerongen G, Bergkamp-Steffens K, Mager WH, Planta RJ: **C-terminal domains of general regulatory factors Abf1p and Rap1p in Saccharomyces cerevisiae display functional similarity**. *Mol Microbiol* 1996, **19**(3):535-543.

11. Remacle JE, Holmberg S: **A REB1-binding site is required for GCN4-independent ILV1 basal level transcription and can be functionally replaced by an ABF1-binding site**. *Mol Cell Biol* 1992, **12**(12):5516-5526.

12. Durocher D, Jackson SP: **The FHA domain**. *FEBS Lett* 2002, **513**(1):58-66.

13. Rudra D, Zhao Y, Warner JR: **Central role of Ifh1p-Fhl1p interaction in the synthesis of yeast ribosomal proteins**. *Embo J* 2005, **24**(3):533-542.

14. Schawalder SB, Kabani M, Howald I, Choudhury U, Werner M, Shore D: **Growth-regulated recruitment of the essential yeast ribosomal protein gene activator Ifh1**. *Nature* 2004, **432**(7020):1058-1061.

15. Martin DE, Soulard A, Hall MN: **TOR regulates ribosomal protein gene expression via PKA and the Forkhead transcription factor FHL1**. *Cell* 2004, **119**(7):969-979.

16. Jorgensen P, Rupes I, Sharom JR, Schneper L, Broach JR, Tyers M: **A dynamic transcriptional network communicates growth potential to ribosome synthesis and critical cell size**. *Genes Dev* 2004, **18**(20):2491-2505.

17. Beer MA, Tavazoie S: **Predicting gene expression from sequence**. *Cell* 2004, **117**(2):185-198.

18. Grigull J, Mnaimneh S, Pootoolal J, Robinson MD, Hughes TR: **Genome-wide analysis of mRNA stability using transcription inhibitors and microarrays reveals posttranscriptional control of ribosome biogenesis factors**. *Mol Cell Biol* 2004, **24**(12):5534-5547.

19. Harbison CT, Gordon DB, Lee TI, Rinaldi NJ, Macisaac KD, Danford TW, Hannett NM, Tagne JB, Reynolds DB, Yoo J *et al*: **Transcriptional regulatory code of a eukaryotic genome**. *Nature* 2004, **431**(7004):99-104.

20. Haugen AC, Kelley R, Collins JB, Tucker CJ, Deng C, Afshari CA, Brown JM, Ideker T, Van Houten B: **Integrating phenotypic and expression profiles to map arsenic-response networks**. *Genome Biol* 2004, **5**(12):R95.

21. Preiss T, Baron-Benhamou J, Ansorge W, Hentze MW: **Homodirectional changes in transcriptome composition and mRNA translation induced by rapamycin and heat shock**. *Nat Struct Biol* 2003, **10**(12):1039-1047.

22. Roepcke S, Zhi D, Vingron M, Arndt PF: **Identification of highly specific localized sequence motifs in human ribosomal protein gene promoters**. *Gene* 2006, **365**:48-56.

23. Yoshihama M, Uechi T, Asakawa S, Kawasaki K, Kato S, Higa S, Maeda N, Minoshima S, Tanaka T, Shimizu N *et al*: **The human ribosomal protein genes: sequencing and comparative analysis of 73 genes**. *Genome Res* 2002, **12**(3):379-390.

24. Tharakaraman K, Bodenreider O, Landsman D, Spouge JL, Marino-Ramirez L: **The biological function of some human transcription factor binding motifs varies with position relative to the transcription start site**. *Nucleic Acids Res* 2008, **36**(8):2777-2786.

25. Zhang Z, Dietrich FS: **Mapping of transcription start sites in Saccharomyces cerevisiae using 5' SAGE**. *Nucleic Acids Res* 2005, **33**(9):2838-2851.

26. Miura F, Kawaguchi N, Sese J, Toyoda A, Hattori M, Morishita S, Ito T: **A large-scale full-length cDNA analysis to explore the budding yeast transcriptome**. *Proc Natl Acad Sci U S A* 2006, **103**(47):17846-17851.

27. Clark TA, Sugnet CW, Ares M, Jr.: **Genomewide analysis of mRNA processing in yeast using splicing-specific microarrays**. *Science* 2002, **296**(5569):907-910.

28. Spingola M, Grate L, Haussler D, Ares M, Jr.: **Genome-wide bioinformatic and molecular analysis of introns in Saccharomyces cerevisiae**. *Rna* 1999, **5**(2):221-234.

29. Planta RJ, Mager WH: **The list of cytoplasmic ribosomal proteins of Saccharomyces cerevisiae**. *Yeast* 1998, **14**(5):471-477.

30. Bhattacharyya N, Banerjee D: **Transcriptional regulatory sequences within the first intron of the chicken apolipoproteinAI (apoAI) gene**. *Gene* 1999, **234**(2):371-380.

31. Chen J, Hayes P, Roy K, Sirotnak FM: **Two promoters regulate transcription of the mouse folylpolyglutamate synthetase gene three tightly clustered Sp1 sites within the first intron markedly enhance activity of promoter B**. *Gene* 2000, **242**(1-2):257-264.

32. Wenz P, Schwank S, Hoja U, Schuller HJ: **A downstream regulatory element located within the coding sequence mediates autoregulated expression of the yeast fatty acid synthase gene FAS2 by the FAS1 gene product**. *Nucleic Acids Res* 2001, **29**(22):4625-4632.

33. Xue W, Wang J, Shen Z, Zhu H: **Enrichment of transcriptional regulatory sites in non-coding genomic region**. *Bioinformatics* 2004, **20**(4):569-575.

34. Zhang J, Hu J, Shi XF, Cao H, Liu WB: **Detection of potential positive regulatory motifs of transcription in yeast introns by comparative analysis of oligonucleotide frequencies**. *Comput Biol Chem* 2003, **27**(4-5):497-506.

35. Zhang KL, Zhang J, Luo JC: **Potential transcriptional synergy between upstream regions and introns of yeast genes**. *Prog Biochem Biophys* 2005, **32**:46-52.

36. Kanhere A, Bansal M: **Structural properties of promoters: similarities and differences between prokaryotes and eukaryotes**. *Nucleic Acids Res* 2005, **33**(10):3165-3175.

37. Zhang J, Shi XF, Yang HF: **Transcription Rates of Yeast Genes Are Influenced by The Distribution of Introns**. *Prog Biochem Biophys* 2003, **30**:945-949.

38. Lieb JD, Liu X, Botstein D, Brown PO: **Promoter-specific binding of Rap1 revealed by genome-wide maps of protein-DNA association**. *Nat Genet* 2001, **28**(4):327-334.

39. Sanchez-Gorostiaga A, Lopez-Estrano C, Krimer DB, Schvartzman JB, Hernandez P: **Transcription termination factor reb1p causes two replication fork barriers at its cognate sites in fission yeast ribosomal DNA in vivo**. *Mol Cell Biol* 2004, **24**(1):398-406.

40. Lang WH, Morrow BE, Ju Q, Warner JR, Reeder RH: **A model for transcription termination by RNA polymerase I**. *Cell* 1994, **79**(3):527-534.

41. Teixeira MC, Monteiro P, Jain P, Tenreiro S, Fernandes AR, Mira NP, Alenquer M, Freitas AT, Oliveira AL, Sa-Correia I: **The YEASTRACT database: a tool for the analysis of transcription regulatory associations in Saccharomyces cerevisiae**. *Nucleic Acids Res* 2006, **34**(Database issue):D446-451.

42. Wysocki R, Fortier PK, Maciaszczyk E, Thorsen M, Leduc A, Odhagen A, Owsianik G, Ulaszewski S, Ramotar D, Tamas MJ: **Transcriptional activation of metalloid tolerance genes in Saccharomyces cerevisiae requires the AP-1-like proteins Yap1p and Yap8p**. *Mol Biol Cell* 2004, **15**(5):2049-2060.

43. Kellis M, Patterson N, Endrizzi M, Birren B, Lander ES: **Sequencing and comparison of yeast species to identify genes and regulatory elements**. *Nature* 2003, **423**(6937):241-254.

44. Christie KR, Weng S, Balakrishnan R, Costanzo MC, Dolinski K, Dwight SS, Engel SR, Feierbach B, Fisk DG, Hirschman JE *et al*: **Saccharomyces Genome Database (SGD) provides tools to identify and analyze sequences from Saccharomyces cerevisiae and related sequences from other organisms**. *Nucleic Acids Res* 2004, **32**(Database issue):D311-314.

45. Lopez PJ, Seraphin B: **YIDB: the Yeast Intron DataBase**. *Nucleic Acids Res* 2000, **28**(1):85-86.

46. Karolchik D, Baertsch R, Diekhans M, Furey TS, Hinrichs A, Lu YT, Roskin KM, Schwartz M, Sugnet CW, Thomas DJ *et al*: **The UCSC Genome Browser Database**. *Nucleic Acids Res* 2003, **31**(1):51-54.

47. Garcia-Martinez J, Aranda A, Perez-Ortin JE: **Genomic run-on evaluates transcription rates for all yeast genes and identifies gene regulatory mechanisms**. *Mol Cell* 2004, **15**(2):303-313.

48. Rahmann S, Müller T, Vingron M: **On the power of profiles for transcription factor binding site detection**. *Statistical Applications in Genetics and Molecular Biology* 2003, **2**(1).

49. Roepcke S, Grossmann S, Rahmann S, Vingron M: **T-Reg Comparator: an analysis tool for the comparison of position weight matrices**. *Nucleic Acids Res* 2005, **33**(Web Server issue):W438-441.

50. Lascaris RF, Mager WH, Planta RJ: **DNA-binding requirements of the yeast protein Rap1p as selected in silico from ribosomal protein gene promoter sequences**. *Bioinformatics* 1999, **15**(4):267-277.

## Tables

**Table 1.** Intron-containing RP genes

| Gene | Expression  level | Transcription rate | Rap1 sites  Abf1, Reb1 sites | IFHL sites |
| --- | --- | --- | --- | --- |
| *RPL21B* | 85.7 | 2.5822 | -241,-225,-125,-121 |  |
| *RPS27B** | 65.6 | 2.4516 | -405,-391 |  |
| *RPS23A* | 52.9 | 2.7043 | -365,-348 | -165(-) |
| *RPL2B* | 54.9 | 1.8372 | -393,-373 |  |
| *RPS21B* | 54.1 | 1.7135 | -287,-270 |  |
| *RPS21A** | 63 | 3.1681 | -196,+105(-) A(-145(-),-111(-)) |  |
| *RPL23A* | 54.2 | 3.8236 | -325,-296 |  |
| *RPS4B* | 45.9 |  | -446,-433 |  |
| *RPL23B* | 50.3 | 2.2774 | -371,-353 | -219,-158(-),-153 |
| *RPS19A* | 45.6 |  | -256,-230 | -444(-),-439,-378 |
| *RPL28* | 50.3 | 12.2799 | -244 | -213,-141,+589(-) |
| *RPS10A* | 40.2 | 2.0841 | -334,-315(-) |  |
| *RPL27A* | 50.1 | 1.885 | -409,-395 | -120, +208(-) |
| *RPL39* | 46 | 7.0498 | -373(-),-339(-) | -216(-),-210,-135(-) |
| *RPL26B* | 44.8 | 1.5293 | -282,-265(-) | -221(-),-179 |
| *RPL43B* | 41.4 |  | -290,-272 |  |
| *RPS19B* | 42.9 | 4.5195 | -367,+79 A(-258,-118(-),-56(-),+35) |  |
| *RPP1B* | 40.8 | 3.863 | -255,-231 |  |
| *RPS30B** | 51.2 | 4.0151 | -426,-210 A(-418(-),-204) | -456,-423(-),-368 |
| *RPL16A* | 27.4 | 2.7637 | -388  A(-18(-),+35) |  |
| *RPS23B* | 42.2 | 2.5263 | -305,-287 |  |
| *RPS30A* | 54.2 |  | -298,-208,-186 | -320, -295(-), -264, +193(-) |
| *RPL33A* | 45.5 | 1.7881 | -352,-336 | -179(-) |
| *RPS18B* | 29.3 | 2.8353 | -378,-353 | -250(-),-238,-185 |
| *RPL35A** | 41.2 | 3.3246 | -273,-257 | -252,-153,+498(-) |
| *RPS17A* | 36.4 | 3.6729 | -332,-319 | -226,-203(-) |
| *RPL17A* | 33.2 | 4.9196 | -346,-328 |  |
| *RPS16B* | 31.2 | 4.0608 | -429(-),-409(-) |  |
| *RPL33B* | 36.5 | 1.5746 | -464  A(-112(-),+26,+62) R(-370(-),-231(-)) |  |
| *RPL14A* | 34.1 | 3.0719 | -373,-354 | -172 |
| *RPL31A** | 45.4 | 2.1804 | -404,-378 | -240(-) |
| *RPL22A* | 39 | 5.2449 | -229,-209 | -434(-),-350(-),+240(-) |
| *RPL42A* | 32.1 | 4.9225 | -351,-331 | -292(-),-249 |
| *RPS18A* | 33.7 | 2.7306 | -307  A(+57(-)) R(+9) |  |
| *RPL34B* | 34.4 | 3.1183 | -447(-),-419(-) | -186 |
| *RPL43A* | 42.3 | 3.7648 | -208  A(-213(-),-203,-134) |  |
| *RPL18A* | 31.5 | 6.4403 | -260(-),-225(-) | -396(-),-299(-),-278, +511(-) |
| *RPL30** | 36.7 | 8.9369 | -298(-),-275(-) | -232(-),-178,-147(-) |
| *RPS6B* | 33.8 |  | -303,-286 |  |
| *RPS17B* | 34.5 | 2.3003 | -289,-265 | -517,-473 |
| *RPL37A* | 32.1 | 2.2123 | -365,-340 |  |
| *RPS7A* | 26.7 | 2.8238 | -474,-449 |  |
| *RPS13** | 28.2 | 7.5174 | -232,-212 | -231(-) |
| *RPL35B* | 32.1 | 3.4986 | -255,-150 | -131(-) |
| *RPL13A* | 25.9 | 3.1935 | -192,-172 |  |
| *RPL40A* | 30.2 | 3.4181 | -348,-329(-) |  |
| *RPS4A* | 25.5 | 2.3058 | -386,-357 |  |
| *RPS6A* | 27.2 | 1.1381 | -430,-405 | -317(-),-294(-),-218(-) |
| *RPL31B* | 24 | 3.476 | -507,-491 |  |
| *RPL27B* | 22.1 | 1.9604 | -329,-314 | -341(-),-267(-) |
| *RPL25* | 28.4 | 5.7814 | -337(-)  A(-399(-),-231(-),-139(-)) |  |
| *RPS9B* | 25.7 | 2.0085 | -201,-185 | -224(-),-149(-) |
| *RPL16B** | 27.1 | 2.4756 | -396,-379 |  |
| *RPL20B* | 25.8 |  | -335  A(-438(-),-361(-)) |  |
| *RPL17B* | 23.7 |  | -507,-493 | -495(-),-277 |
| *RPS27A** | 26.6 | 2.1075 | -196,-175 |  |
| *RPL7A* | 26.7 | 2.8021 | -232,-214 |  |
| *RPS14A** | 33.5 | 4.7763 | -261(-),-244 |  |
| *RPL42B* | 24.4 | 4.4354 | -357,-340,-319 |  |
| *RPS24A* | 24.5 | 2.0866 | -313  A(-245,-221(-)) | -336(-) |
| *RPL19A* | 25.1 | 2.7631 | -414,-398 | -245 |
| *RPS24B* | 23.1 | 3.8824 | -450  A(-280,-211(-)) |  |
| *RPL19B** | 23.6 | 2.8289 | -370,-353 |  |
| *RPL13B* | 19.4 | 3.1281 | -337,-322 |  |
| *RPL34A* | 17.2 | 4.0404 | -345(-),-306(-) |  |
| *RPS0B* | 16.6 | 2.1791 | -394,-373 | -224(-),-180(-) |
| *RPS0A* | 17.1 | 1.3836 | -485,-467 |  |
| *RPL6A* | 19.3 | 1.5821 | -309,-290(-) |  |
| *RPS16A* | 15.8 |  | -305,-289 | -362(-),-307 |
| *RPS7B* | 14.4 | 1.795 | -287,-271 |  |
| *RPS10B* | 17.5 | 1.0443 | -192,-177 | -262 |
| *RPS11B* | 15 | 1.6542 | -288,-272 | -322,-277(-),+551,+250(-) |
| *RPL36A** | 14 | 2.195 | -146,-58(-)  A(-321) | -318(-),-276(-) |
| *RPL7B* | 14.3 | 1.4915 | -193  A(-302,-21,+10) | -294 |
| *RPL26A* | 9.4 | 2.8502 | -293,-251(-) |  |
| *RPS22B* | 13.6 | 3.5108 | A(+62,+87(-)) | -204(-) |
| *RPS14B* | 7.9 |  | -534(-),-185  A(-595,+34) | -162(-) |
| *RPS9A** | 4.5 | 8.3216 | -204,+226 |  |
| *RPL2A* |  | 5.2272 | -486  A(-430) R(-486(-)) |  |
| *RPL22B* |  |  | -206,-183 |  |
| *RPL36B* |  |  | -279  R(-279(-)) | -144(-) |
| *RPS29B* | 50.1 | 10.2652 | -431(-),-204 A(-493(-),-117(-)) | -387(-),-364(-) |
| *RPL20A* | 15.4 | 3.2788 | -136(-), +293(-)  A(+312(-)) |  |
|  |  |  |  |  |
| *RPL24A** | 50.1 | 3.7488 | -355,-332 | -258(-),-203,-170(-),+124 |
| *RPL24B* | 55.8 | 4.8691 | -341,-324 | -415,-112 |
| *RPS8B* | 47.3 | 3.3601 | -389,-366 | -250 |
| *RPS25A** | 50.1 | 3.6055 | -213,+16(-)  A(-188(-),-27) |  |
| *RPS25B** | 50.1 |  | -248  A(-462,-405(-)) |  |
| *RPS26B* | 50.1 | 6.5558 | -299 A(-496,-413,-131,-19(-)) | +549(-) |
| *RPL32* | 37 | 13.2378 | -444,-425(-) | -204(-),-182(-) |

The last 7 genes contain introns in their 5’UTR (leader intron). The second and third columns denote expression level (<http://web.wi.mit.edu/young/expression>) and transcription rate [47], respectively. The fourth column lists Rap1 sites. For genes without the typical duplicate Rap1 sites (see text) or single Rap1 sites, potential Abf1 and Reb1 sites are shown. (“A” denotes Abf1 and “R” Reb1). Asterisks mark genes with newly identified canonical Rap1 sites. The minus in brackets indicates the orientation of the motif.

**Table 2.** Intronless RP genes

| Gene | Expression  level | Transcription  rate | Rap1 sites  Abf1, Reb1 sites | IFHL sites |
| --- | --- | --- | --- | --- |
| RPL11B | 52 | 13.4009 | -232, -217 |  |
| *RPP2A* | 49.4 | 10.8996 | -321, -289(-) |  |
| *RPS22A* | 50.1 | 10.7089 | -255, -221 | -159 |
| *RPS20* | 50.1 | 4.9583 | -230, -213 | -291(-) |
| *RPS28A* | 50.1 | 35.889 | -484  A(-153, -111) |  |
| *RPS28B* | 50.1 | 25.5955 | A(-143, -15) |  |
| *RPS31* | 52.7 | 21.8617 | -218  A(-54) R(-534(-),+80,+93(-)) |  |
| *RPS5** | 37.4 | 10.2932 | -445, -420 | -492(-) |
| *RPL1B* | 61.7 | 6.3814 | -250, -230(-) | -155(-) |
| *RPP2B* | 51.2 | 15.6469 |  |  |
| *RPL10* | 55.5 | 20.0518 | -250, -233 | +336, +420(-) |
| *RPL9A* | 34.5 | 12.5946 | -296(-)  A(-426(-), +87) |  |
| *RPL5* | 37 |  | -271,-253 |  |
| *RPL4A* | 44.6 | 2.7488 | A(-474, -166(-)) | +249(-) |
| RPL1A* | 44.8 | 4.717 | -136(-)  A(-227(-), -148(-)) |  |
| *RPL3* | 36.7 | 6.0533 | A(-281(-),-210(-)) |  |
| *RPL38* | 36.7 |  | -269, -251 |  |
| *RPS15* | 30.6 | 11.7827 | -362, -330(-) |  |
| *RPL11A* | 34.5 | 9.8776 | -357, -340(-) |  |
| *RPS12* | 35.6 | 10.2392 | -227, -201 |  |
| *RPS1A* | 31 | 4.2657 | -220  A(-239(-)) |  |
| *RPP1A* | 34.2 |  | A(-515(-), -410(-)) |  |
| *RPL12B* | 23.1 | 8.5492 | -482, -465(-) | -289, -255(-) |
| *RPL15A* | 24.1 | 8.1666 | -212(-),-78  A(-246) | -195 |
| *RPL8B* | 23.5 | 5.7545 | -265 A(-171,-154(-),-75(-)) |  |
| *RPL4B* | 24.7 | 3.6823 | A(-296,-157(-)) R(-380(-)) |  |
| *RPP0* | 24.6 | 4.5681 | -205 A(-171,-154(-),-75(-)) | +336, +420(-) |
| *RPL12A* | 18.6 | 8.2067 | -504, -487(-) |  |
| *RPL9B** | 14.6 | 13.6891 | -241(-)  A(-334,+78) |  |
| *RPS1B* | 11.2 | 4.8297 | -373, -343 |  |
| *RPS3* |  | 7.2458 | -232 |  |
| *RPL41A* |  |  | -256,+384 A(+12(-),+218(-)) R(-145(-),-140(-),-122(-)) |  |
| *RPL41B** |  |  | -329,-321,-305 |  |

For table description see Table 1.

## Figure Legends

**Figure 8.** Profile of GC base content (Additional file 8)

The promoters of each gene set are aligned at the TSS, which is indicated with a vertical line. (A) intron-containing RP genes (*N*=90), (B) intronless RP genes (*N*=33), and (C) lowly expressed intron-containing genes (*N*=35). For each set, the GC-content at each position is depicted and a smoothed curve is drawn using the Lowess method of the R-package.

**Figure 9.** Distributions of binding site motifs for several transcription factors (Additional file 9)

Each chart depicts the distribution of the motif occurrence of one factor in the three different promoter sets. The factors are indicated by their names in the upper left corner of each chart.
