# Supplementary material for: Characteristic differences between the promoters of intron-containing and intronless ribosomal protein genes in yeast
Source: BMC Res Notes. 2008 Oct 29;1:109. doi: 10.1186/1756-0500-1-109 (PMC2585575; doi:10.1186/1756-0500-1-109)
Supplement: Additional File 5 — Promoter regions of RPS29A and B. A snapshot from the Genome Browser illustrates a typical the structure of RP gene promoters. [file 1756-0500-1-109-S5.pdf]

Supplementary Figure S4

Genome Browser snapshots of the promoter regions of RPS29A and B of *Saccharomyces cerevisiae* (Karolchik et al., 2003)

RPS29A

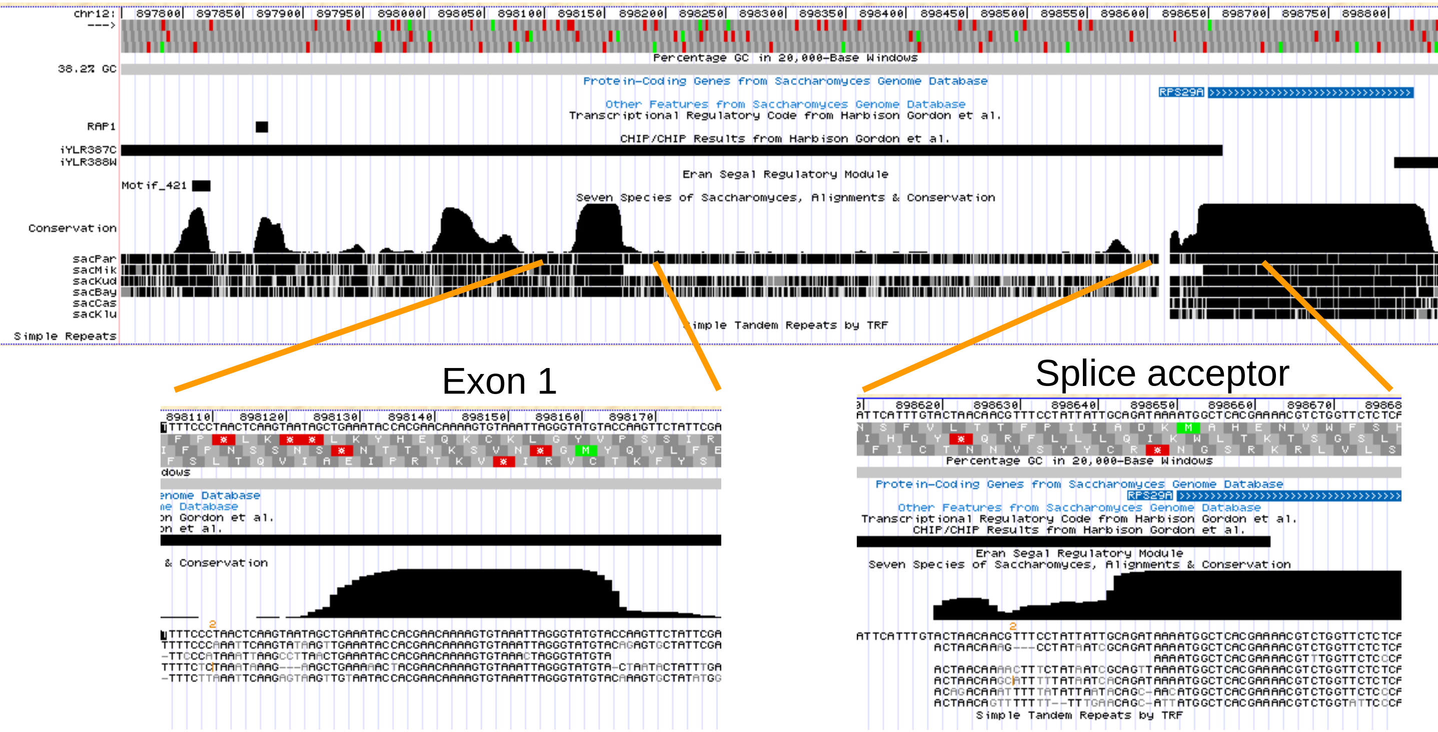

RPS29B (reverse strand)

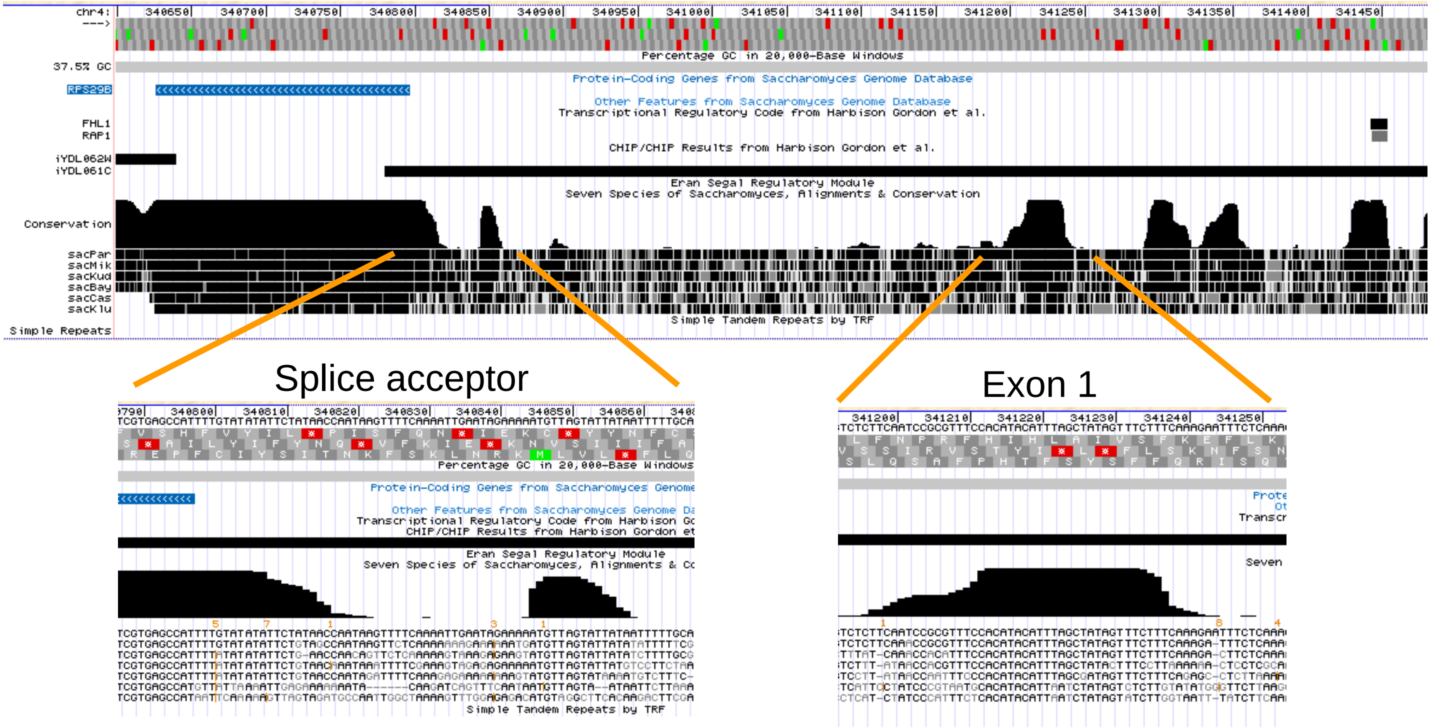

Figure S4 Promoter architecture of RPS29A and RPS29B

We illustrate the typical RP promoter architecture with the paralogous genes *RPS29A* and *RPS29B*, which are characterized as intronless in the RPG database and in the UCSC Genome Browser but as intron-containing in SGD and in Seraphin's intron table (Christie, et al., 2004; Karolchik, et al., 2003; Lopez and Seraphin, 2000; Miura, et al., 2006; Nakao, et al., 2004). According to the 5'SAGE data, the TSS of *RPS29B* is localized 452 base pairs upstream of the ATG. The typical 5'UTRs span 10 to 40 nucleotides and the typical intron size in RP genes is approximately 400 nucleotides (Fig. 1A), supporting the existence of a leader intron in its 5'UTR. Additional evidence comes from the pattern of evolutionary sequence conservation, existing splice site consensi, and a potential Rap1 binding site at the proper location upstream of the predicted non-coding exon. Although not corroborated by 5'SAGE data, similar observations hold true for the paralogous gene *RPS29A*.
