# Supplementary figures and images for: Characteristic differences between the promoters of intron-containing and intronless ribosomal protein genes in yeast
Source: BMC Res Notes. 2008 Oct 29;1:109. doi: 10.1186/1756-0500-1-109 (PMC2585575; doi:10.1186/1756-0500-1-109)

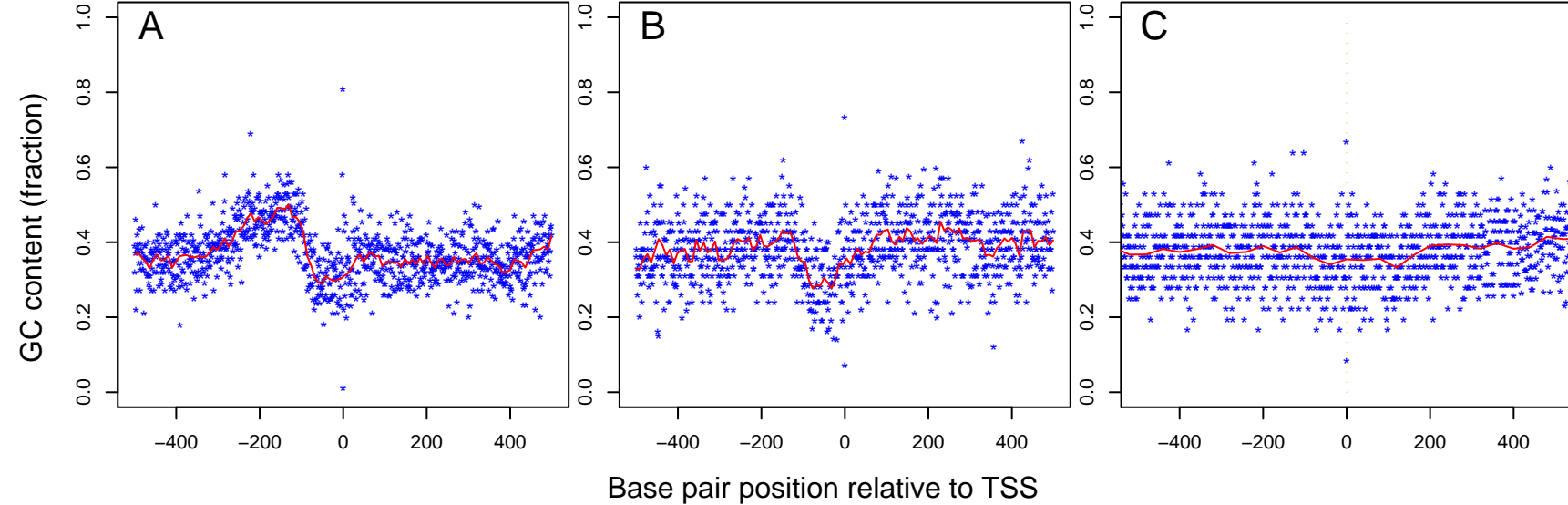

Supplement: Additional File 8 — Profile of GC base content. The profile of GC base content is specifically optimized in the highly expressed RP genes. [file 1756-0500-1-109-S8.pdf]

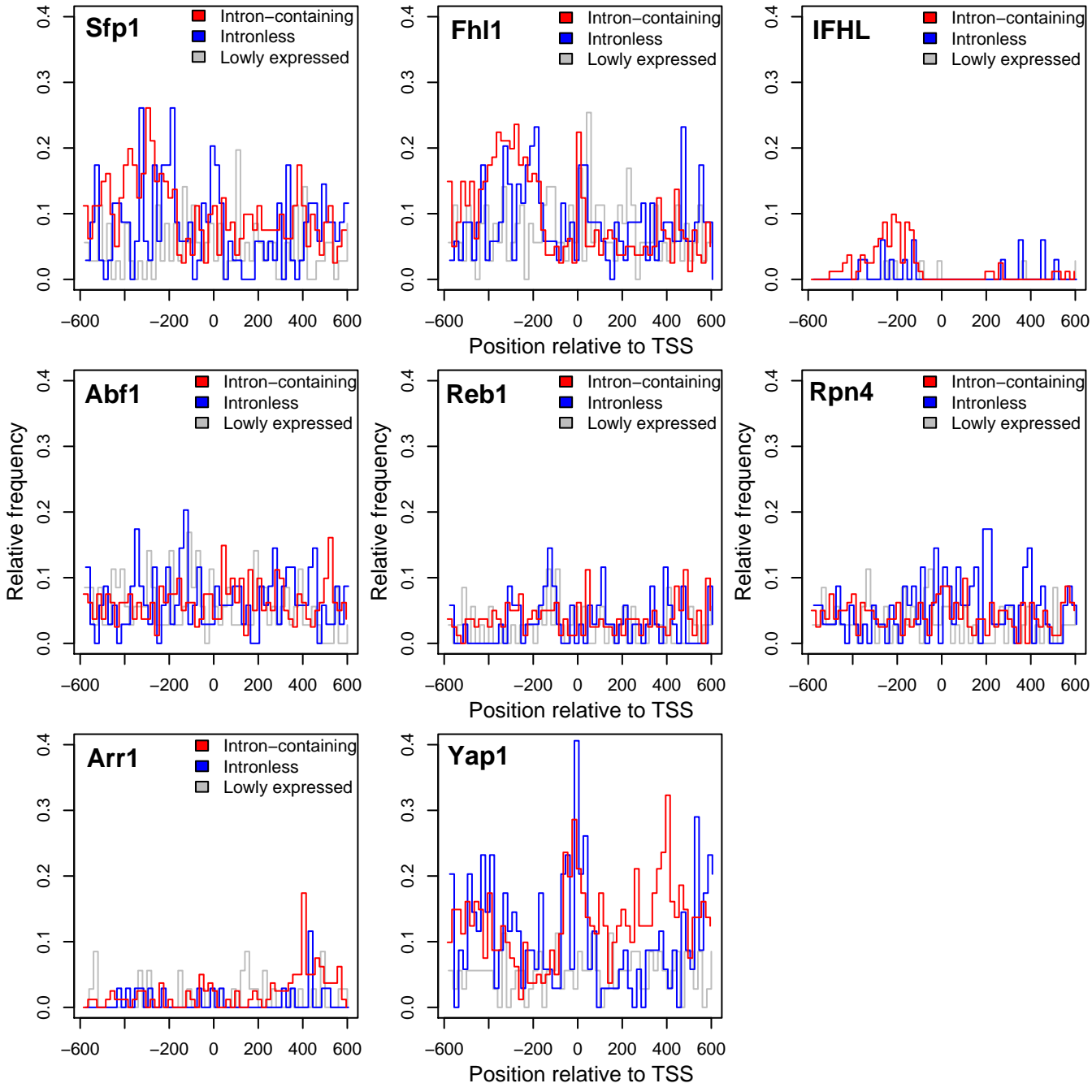

Supplement: Additional File 9 — Motif hit distributions. Distributions of binding site motifs for several transcription factors. [file 1756-0500-1-109-S9.pdf]
